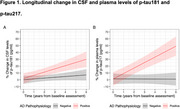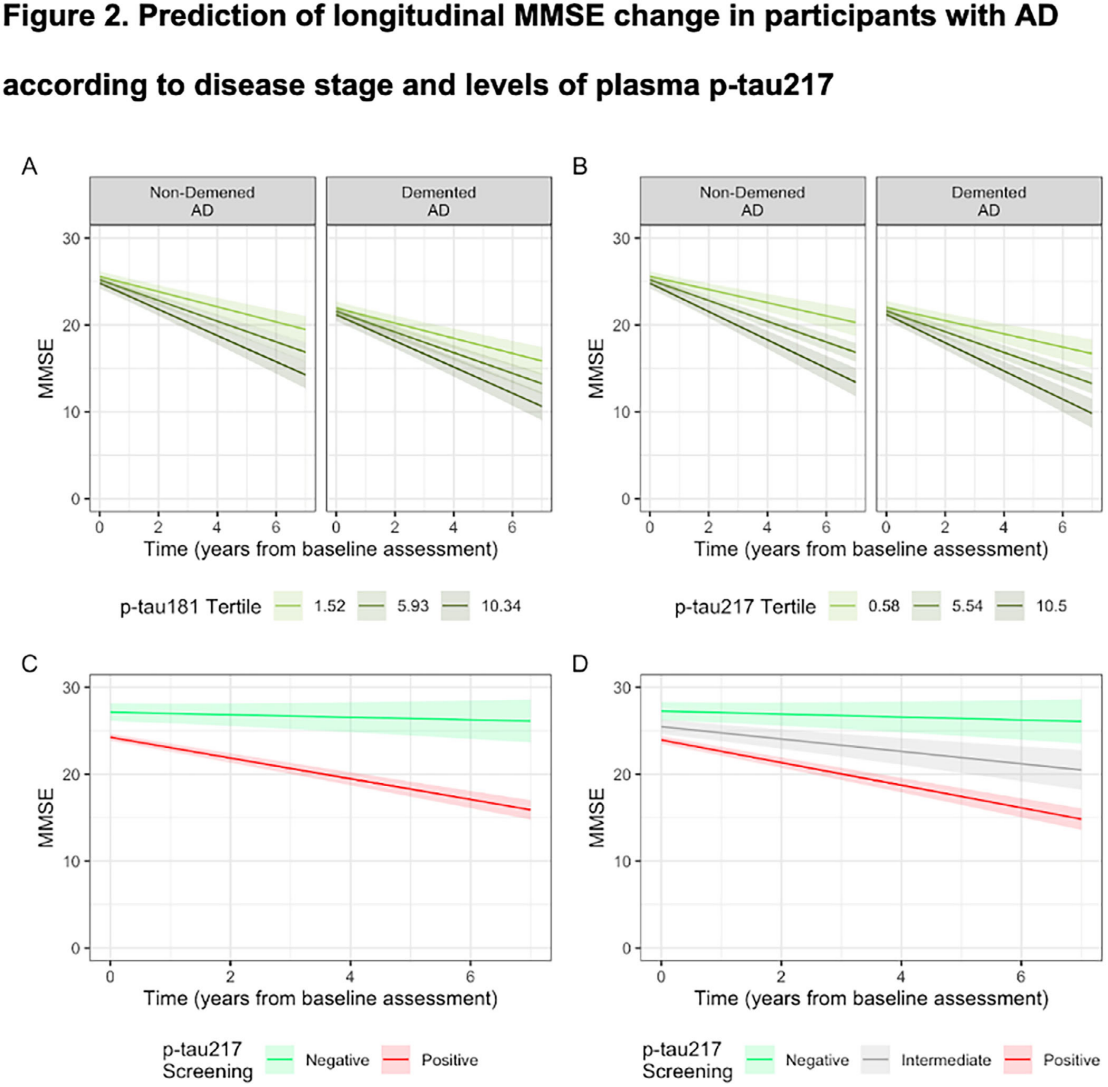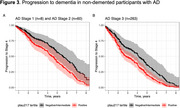# Plasma Phosphorylated Tau 217 predicts cognitive and functional deterioration across clinical stages of Alzheimer's disease

**DOI:** 10.1002/alz70856_100198

**Published:** 2025-12-25

**Authors:** Judit Selma‐Gonzalez, Sara Rubio‐Guerra, Jesús Garcia Castro, Elena Vera, Isabel Sala, María Belén Sánchez‐Saudinós, Nuole Zhu, Javier Arranz, José Enrique Arriola‐Infante, Íñigo Rodríguez‐Baz, Lucía Maure‐Blesa, Oriol Dols‐Icardo, Laura Videla, Sílvia Valldeneu, Isabel Barroeta, Miguel A Santos‐Santos, Maria Carmona‐Iragui, Lídia Vaqué‐Alcázar, Esther Álvarez‐Sánchez, Oriol Lorente, Mireia Carreras, Olivia Belbin, Burak Arslan, Nicholas J. Ashton, Henrik Zetterberg, Kaj Blennow, Laia Montoliu‐Gaya, Alexandre Bejanin, Alberto Lleo, Juan Fortea, Daniel Alcolea, Ignacio Illán‐Gala

**Affiliations:** ^1^ Sant Pau Memory Unit, Hospital de la Santa Creu i Sant Pau ‐ Biomedical Research Institute Sant Pau ‐ Universitat Autònoma de Barcelona, Barcelona, Spain; ^2^ CIBERNED, Network Center for Biomedical Research in Neurodegenerative Diseases, National Institute of Health Carlos III, Madrid, Spain; ^3^ Sant Pau Memory Unit, Hospital de la Santa Creu i Sant Pau ‐ Biomedical Research Institute Sant Pau ‐ Universitat Autònoma de Barcelona, Barcelona, Barcelona, Spain; ^4^ Sant Pau Memory Unit, Hospital de la Santa Creu i Sant Pau ‐ Biomedical Research Institute Sant Pau ‐ Universitat Autònoma de Barcelona, Barcelona, Cataluña, Spain; ^5^ Sant Pau Memory Unit, Hospital de la Santa Creu i Sant Pau, Institut de Recerca Sant Pau ‐ Universitat Autònoma de Barcelona, Barcelona, Spain; ^6^ Barcelona Down Medical Center, Fundació Catalana Síndrome de Down, Barcelona, Spain; ^7^ Department of Medicine, Faculty of Medicine and Health Sciences, Institute of Neurosciences, University of Barcelona, Barcelona, Spain. Institut d’Investigacions Biomèdiques August Pi i Sunyer (IDIBAPS), Barcelona, Spain; ^8^ Department of Psychiatry and Neurochemistry, Institute of Neuroscience and Physiology, The Sahlgrenska Academy, University of Gothenburg, Mölndal, Gothenburg, Sweden; ^9^ Banner Alzheimer's Institute and University of Arizona, Phoenix, AZ, USA; ^10^ Department of Psychiatry and Neurochemistry, Institute of Neuroscience and Physiology, The Sahlgrenska Academy, University of Gothenburg, Mölndal, Sweden

## Abstract

**Background:**

Phosphorylated tau at threonine 217 (*p*‐tau217) is a highly specific blood‐based biomarker for Alzheimer's disease (AD) pathology, with high diagnostic accuracy and reproducible cut‐offs across cohorts. However, the prognostic utility of plasma *p*‐tau217 has not been assessed across the clinical stages of AD. The objectives of this study were to evaluate the prognostic utility of a commercially available immunoassay for plasma *p*‐tau217 in predicting clinical and functional decline across the clinical stages of AD in a cohort with up to 10 years of follow‐up.

**Method:**

This cohort study analyzed data from the Sant Pau Initiative on Neurodegeneration (SPIN) cohort, including baseline visits conducted between March 2011 and November 2022. Participants included individuals with and without cognitive impairment, classified into clinical stages 1–6 based on AD pathology status in cerebrospinal fluid (CSF), determined by the *p*‐tau181/Aβ1‐42 ratio. Plasma *p*‐tau217 concentrations were measured using a commercially available immunoassay (ALZpath pTau217 assay). Cognitive and functional decline were assessed via changes in the Mini‐Mental State Examination (MMSE) and progression to stage 4. Longitudinal plasma *p*‐tau217 changes were also analyzed according to baseline AD pathology status.

**Result:**

The study included 731 participants (mean [SD] age, 71.5 [10.1] years; 442 females [60%], 289 males [40%]). Among individuals with AD pathology, plasma *p*‐tau217 levels (but not CSF *p*‐tau181) increased with advancing clinical stages, and longitudinal changes in plasma *p*‐tau217 exhibited greater annual increases than CSF *p*‐tau181. Both plasma *p*‐tau217 levels and CSF *p*‐tau181 correlated with cognitive measures and predicted faster cognitive decline. Based on established cut‐offs, participants with negative or intermediate *p*‐tau217 levels experienced slower cognitive and functional decline than those with positive plasma *p*‐tau217 levels. In non‐demented participants, plasma *p*‐tau217 (but not CSF *p*‐tau181) was independently associated with accelerated progression to the dementia stage.

**Conclusion:**

Plasma *p*‐tau217, measured using a commercially available immunoassay, was associated with cognitive and functional decline in AD. These findings highlight the potential of plasma *p*‐tau217 for use in routine clinical practice to monitor and prognosticate AD progression.